# Multilayer Epitaxial Graphene on Silicon Carbide: A Stable Working Electrode for Seawater Samples Spiked with Environmental Contaminants

**DOI:** 10.3390/s20144006

**Published:** 2020-07-18

**Authors:** Lisa C. Shriver-Lake, Rachael L. Myers-Ward, Scott N. Dean, Jeffrey S. Erickson, David A. Stenger, Scott A. Trammell

**Affiliations:** U.S. Naval Research Laboratory, 4555 Overlook Avenue SW, Washington, DC 20375, USA; lisa.shriverlake@nrl.navy.mil (L.C.S.-L.); rachael.myers-ward@nrl.navy.mil (R.L.M.-W.); scott.dean@nrl.navy.mil (S.N.D.); jeffrey.erickson@nrl.navy.mil (J.S.E.); david.stenger@nrl.navy.mil (D.A.S.)

**Keywords:** multilayer epitaxial graphene, cyclic square wave voltammetry, seawater, identification algorithms, machine learning

## Abstract

The electrochemical response of multilayer epitaxial graphene electrodes on silicon carbide substrates was studied for use as an electrochemical sensor for seawater samples spiked with environmental contaminants using cyclic square wave voltammetry. Results indicate that these graphene working electrodes are more robust and have lower background current than either screen-printed carbon or edge-plane graphite in seawater. Identification algorithms developed using machine learning techniques are described for several heavy metals, herbicides, pesticides, and industrial compounds. Dose-response curves provide a basis for quantitative analysis.

## 1. Introduction

The development of miniaturized and low power electrochemical sensors [1] for deployment into the ocean [2,3,4,5,6,7] is of interest to the U.S. Navy. For example, water monitoring for industrial contaminants such as polyaromatic hydrocarbons and other toxic pollutants is important for diver safety [8]. Commonly used electrochemical methods, including square wave voltammetry, are broad-spectrum detection techniques, but many unattended field experiments are run without sample preparation or separation steps and simply monitor for changes. In these cases, post-collection data analysis procedures must be performed on the voltammograms to resolve mixtures and identify peaks.

It has been shown that machine and deep learning models are capable of classifying samples based on their electrochemical signatures. A previous study [9] evaluated the ability of several different machine learning methods to identify nitrogen-containing compounds as well as various environmental contaminants in seawater. This work was performed with screen-printed carbon electrodes due to their low cost and availability. While the results of the study were promising, the limits of detection could be greatly improved through the use of a more sensitive electrode. Furthermore, there is some concern regarding the ability of any screen-printed electrode to survive a deployment in submerged conditions, a situation that might reasonably be expected for even short-term unattended monitoring.

A working electrode deployed in seawater must be robust and have a large potential window with a practical sensitivity for a variety of analytes. Traditional metal electrodes like Pt and Au have a limited potential window in water [10], while glassy carbon can have a high capacitance affecting sensitivity [11], which can be improved with chemical modification [12]. There has been recent interest in the use of graphene for electrochemical detection. Graphene is a promising material for the fabrication of highly sensitive working electrodes; it has unique physical and electrical properties that have been reviewed for a variety of electroanalytical applications [13,14,15,16].

It is hypothesized that multilayer epitaxial graphene on silicon carbide (SiC) will be a stable working electrode for cyclic square wave voltammetry in seawater. Previous work from this group demonstrated an increased sensitivity for the detection of 2,4,6-trinitrotoluene (TNT) over conventional screen-printed carbon electrodes using plasma-modified, epitaxial graphene [17]. The goal of this work is to extend that effort by demonstrating detection of several heavy metals, herbicides, pesticides, and industrial compounds in seawater. Chemical identification algorithms developed using machine learning techniques are reported. While previous machine learning studies did not attempt to assign concentrations to identified species [9], dose-response curves are explored in this work as the basis of a quantitative analysis.

## 2. Materials and Methods

### 2.1. Chemicals, Seawater and Electrodes

Heavy metals and other chemicals were purchased from Sigma-Aldrich (St. Louis, MO, USA) including CuSO_4_, PbCl_2_, HgCl_2_, CdCl_2_, Diquat dibromide (DQBr_2_), Paraquat dichloride (PQCl_2_), methyl parathion (MeP), and bisphenol A (BPA). These chemicals were used to prepare 1 mg/mL (1000 ppm) stock solutions. Most of the solutions were prepared in 18 mΩ water with the exceptions of MeP and BPA. MeP was prepared in methanol, while 1 drop of concentrated sodium hydroxide was added to the BPA-water solution to make it basic, thereby dissolving the BPA. For these studies, the U.S. Naval Research Laboratory in Key West, FL, USA provided ocean water. Test samples were prepared by adding either 1, 5, 10, or 20 µL of the 1 mg/mL stock solution into a vial containing 10 mL of Key West ocean water. The final concentrations were 100, 500, 1000, or 2000 ppb, respectively.

Screen-printed carbon electrodes (SPE) with a geometric area = 0.20 cm^2^ and edge-plane graphite electrodes (EPG) with a geometric area = 0.20 cm^2^ were obtained from Pine Research Instrumentation (Durham, NC, USA). Multilayer graphene electrodes (exposed geometric area = 0.80 cm^2^) were made in-house as described below. For the EPG and graphene studies, a compact spiral platinum counter electrode (99.9% Pt) and an Ag/AgCl reference electrode were used (Metrohm, Redox.me, Riverview, FL, USA). The SPE contained the counter and reference electrodes.

### 2.2. Multilayer Epitaxial Graphene

**Figure 1:**
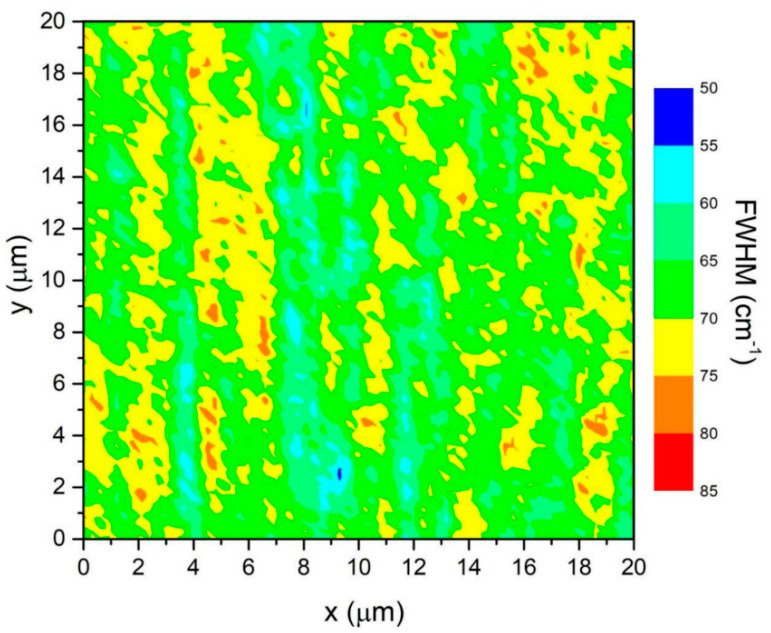
A map of the full width at half maximum (FWHM) of the 2D Raman peak for multilayer graphene.

Multilayer epitaxial graphene was synthesized from a conducting N^+^, Si-face, 4° off-axis towards the [11,12,13,14,15,16,17,18,19,20], 4H^−^ silicon carbide (SiC) substrate via Si sublimation. The synthesis took place in a commercial chemical vapor deposition reactor at a temperature of 1550 °C and a pressure of 100 mbar in an Ar ambient atmosphere [18]. A Thermo DXRxi Raman Microscope was used to map (20 µm^2^) the full width at half maximum (FWHM) of the 2D peak using a 532 nm laser at 9.6 mW and a spot size of 0.3 µm (100 × objective). The map is shown in Figure 1. A growth time of 25 min enabled the graphene thickness to be 2–3 monolayers, where the FWHM of the 2D peak ranged from 50–80 cm^–1^, with the majority being between 60–75 [19].

### 2.3. Electrochemistry

Custom-built potentiostat (the CStat) was used for all assays reported in this work. The CStat has been previously demonstrated for electrochemical detection of multiple compounds, including nitrogen-containing explosives, heavy metals, herbicides, pesticides, and industrial chemicals [1,9]. Three different sample holders were employed for the electrochemical analysis. The holder used for graphene was a custom-made Teflon cell attached to the rigid electrode using a gasket and clamp. The upper funnel-shaped well allowed the counter and reference electrodes to be suspended in the test sample. The SPE was immersed into a scintillation vial containing the 10 mL sample. For EPG, the electrode was lowered into a glass conical container with the working and counter electrodes.

Each 10 mL sample was placed into the holder, such that all three electrodes (working, reference, and counter) were in contact with the solution without touching each other. Cyclic square wave voltammetry was programed to have the voltage sweep from +1.0 to −1.0 and back to +1.0 V vs. Ag/AgCl with step size of 4 mV. A two-minute accumulation step took place at the initial +1.0 V and then again at −1.0 V vs. Ag/AgCl. The square wave frequency was 17.5 Hz with 25 mV amplitude using a current range for the instrument set to 200 μA. At the start of each session, a blank Key West water sample was measured to provide a background for the analysis. Each compound concentration was measured four times.

### 2.4. Machine Learning

Machine learning identification algorithms and library development were repeated as before from our previous paper using Long Short-Term Memory (LSTM) and Fully Convolutional Networks (FCNs) for classification of compounds [9]. The ALSTM-FCN model described by Dean et al. [9] was used for classification unless otherwise noted. Receiver Operating Characteristic (ROC) curves, and ROC area under the curve (AUC) values, were calculated from the Sci-Kit Learn library metric module using the classification report, ROC curve, and AUC functions [20]. Class Activation Maps (CAMs) were plotted where values from the final 1D convolutional layer were visualized as heat-maps plotted over the original input. Curve fitting was performed using the nonlinear least squares function in R stats package [21]. Type of dose response curve used (linear, sigmoidal, or hyperbolic) was selected by lowest mean standard error of the fits. Train-test splits of 70:30 were used for both ML model training and for generating fits used for concentration prediction.

## 3. Results and Discussion

**Figure 2:**
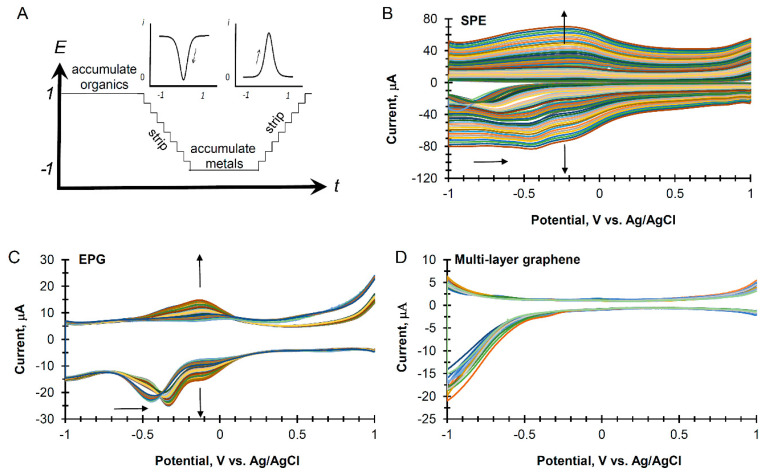
(**A**). Parameters used for cyclic square wave voltammetry including two accumulation steps at two minutes each, a square wave frequency of 17.5 Hz, and a potential range from +1.0 to −1.0 V vs. Ag/AgCl. (**B**,**C**). The continuous scanning of cyclic square wave voltammetry in unspiked seawater using (**B**) a screen-printed carbon electrode (SPE), (**C**) an edge-plane graphite electrode, and (**D**) a multilayer graphene electrode.

The parameters used for the cyclic square wave voltammetry are shown in Figure 2A. The technique involved an in situ cleaning of the electrode immediately before use, adsorption of the analyte, and stripping voltammetry for detection. Example cyclic square wave voltammograms (CSWV) of unspiked seawater from West Florida using different electrodes are shown in Figure 2B–D.

The most common feature in the CSWVs is a cathodic peak for the reduction of oxygen. As shown in Figure 2B, using a carbon SPE, the initial peak occurred near −0.9 V vs. Ag/AgCl but drifted to more positive values with continued scans. Additionally, SPE electrodes also increased in the capacitance current upon further scanning in seawater with a set of new peaks centered at −0.2 V vs. Ag/AgCl. In Figure 2C, the results of continued scanning using CSWV with edge-plane graphite electrodes gave a cathodic peak for the reduction of oxygen near −0.5 V vs. Ag/AgCl, which also drifted to more positive values with continued scans. Upon further scanning in seawater, a new set of peaks formed at −0.1 V. In contrast, Figure 2D shows that multilayer graphene suppressed the reduction of oxygen. The electrode displayed a very stable and low capacitance current with no additional peaks growing in with excess scanning.

### 3.1. Multilayer Graphene

**Figure 3:**
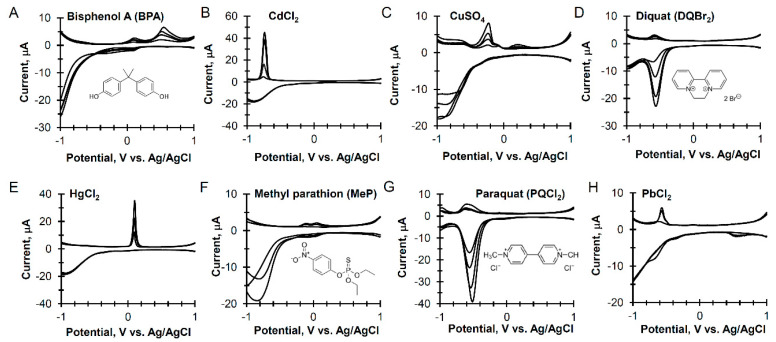
Cyclic square wave voltammetry of seawater spiked with environmental contaminants with increasing concentration from 100 to 2000 ppb using a multilayer graphene as the working electrode and parameters listed in Figure 2A.

Graphene electrodes were further studied using seawater samples spiked with one of four types of analytes: heavy metals, herbicides, pesticides, and industrial compounds. The same CSWV procedure shown in Figure 2A was used for these experiments. In Figure 3, the distinctive electrochemical signatures for each class of compounds are shown at concentrations of 100, 500, 1000, and 2000 ppb.

BPA has two anodic peaks, one at 0.12 V and a larger peak at 0.54 V vs. Ag/AgCl. For the various heavy metal cations, the cathodic peaks are concomitant with oxygen reduction, and the anodic stripping peaks have peak potentials at −0.76 V (Cd), −0.23 V (Cu), 0.11 V (Hg), and −0.57 V (Pb), vs. Ag/AgCl. The reduction of the herbicides, DQBr_2_ and PQCl_2_, show cathodic peaks at −0.54 V vs. Ag/AgCl, with small anodic broad peaks centered at −0.58 V (DQBr_2_), and 0.60 V (PQCl_2_) vs. Ag/AgCl.

**Figure 4:**
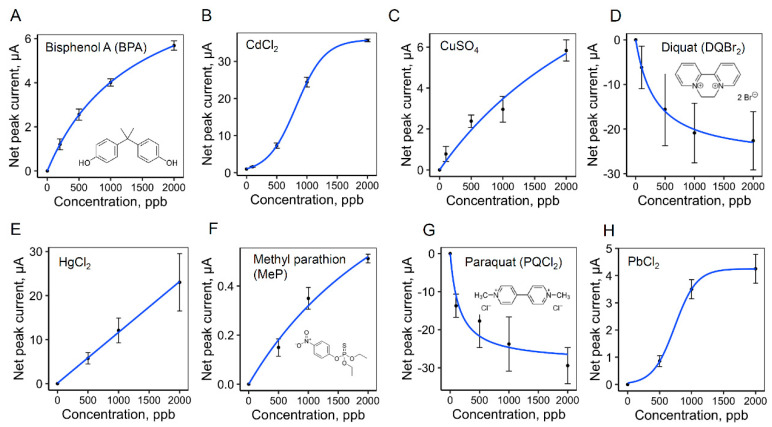
The dose response of seawater spiked with environmental contaminants. The peaks from the cyclic square wave voltammetry and fitting parameters used for the analysis are listed in Table 1.

Using a distinctive peak for each analyte from the cyclic square wave voltammograms, the dose response in current between 100–2000 ppb for each analyte is plotted in Figure 4. Two of the heavy metals, CdCl_2_ and PbCl_2_, displayed a sigmoidal response (Equation (1)). The response for CuSO_4_ was hyperbolic (Equation (2)), and HgCl_2_ was linear. The dose responses of DQBr_2_, PQCl_2_, MeP, and BPA were all hyperbolic. The electrochemical peak positions, fitting parameters used for the analysis and estimated limits of detection (LODs), are listed in Table 1.

f = a/(1 + exp(−(x − x_0_)/b)) (1)

f = a*x/(1 + b*x)(2)

A curved hyperbolic response can be explained by a Langmuir binding isotherm at the electrode surface [10], in which the analyte has an interaction with an active site at the graphene electrode surface described by a formation constant, i.e., related to parameter b, in Equation (2). The sigmoidal responses for Cd and Pb are harder to explain. One possible description consistent with the observation is that at low metal ion concentration, the active sites at the graphene electrode poorly reduce the metal ion. At medium concentrations, more of the metal is reduced, which makes for a better nucleation site for further reduction and increased signal. At the highest concentrations, all the sites become saturated and the response levels turned off.

### 3.2. Identification Using Machine Learning

**Figure 5:**
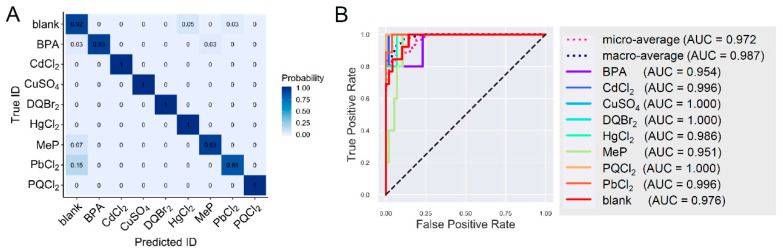
The confusion matrix (**A**) and Receiver Operating Characteristic (ROC) curves (**B**) for the library of cyclic square wave voltammetry of seawater spiked with environmental contaminants using a multilayer graphene as the working electrode.

The previous strategy for data processing was used prior to training for the machine learning analysis, which included the concatenation of cathodic and anodic scans for each sample, but otherwise the experimental data was not modified [9]. The confusion matrix for the complete set of data well as the ROC curves and area under the ROC curves (AUC) are shown in Figure 5. Most of the compounds have a high probability of the correct identification (>0.9), except for PbCl_2_, which was 0.85. All of the compounds have AUCs > 0.90.

**Figure 6:**
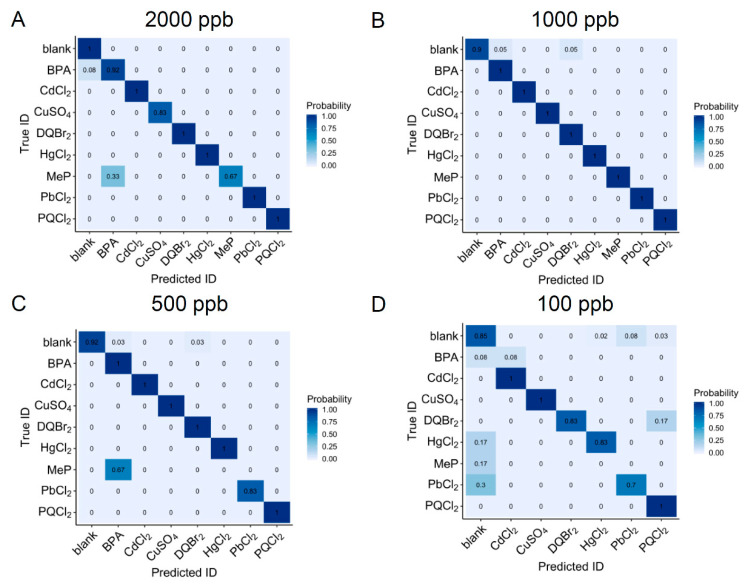
The confusion matrix at each concentration. (**A**) 2000 ppb, (**B**) 1000 ppb, (**C**) 500 ppb, and (**D**) 100 ppb.

The ability of the identification algorithm to correctly ID the true compound begins to degrade at lower concentrations, as shown in Figure 6. At 2000 and 1000 ppb, all of the compounds are predicted with high probability (>80%), except for MeP, which is reasonable based on the estimated LODs listed in Table 1. At 500 ppb, MeP is no longer identified, and at 100 ppb, both MeP and BPA are no longer identified by the ALSTM-FCN, most likely due to the lower signal amplitude in the electrochemical signature. The low probability scores for MeP (0), PbCl_2_ (0.7), and HgCl_2_ (0.83) at 100 ppb are also reflected in their poor LODs in the dose response curves.

**Figure 7:**
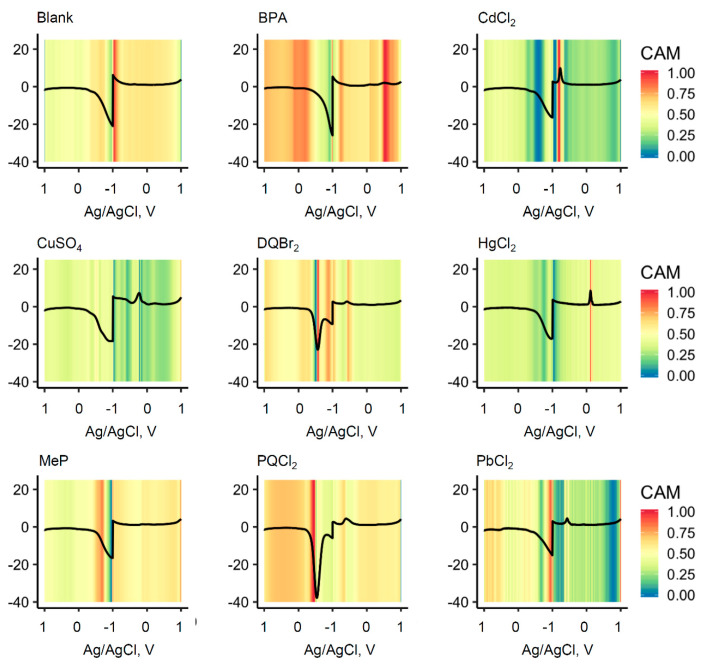
Class activation maps. Representative class activation maps (CAMs) of the compounds in the study.

The FCN-containing models used in the study allow for visualization of the class activation map (CAM). As shown in Figure 7, example CAMs generated by the algorithm identified the major peaks and valleys in the electrochemical signature of each compound used for its identification. The plots are displayed with the potential axis concatenated. For all of the heavy metals tested, the anodic striping peak was clearly the most prominent feature used in the algorithm. For CuSO_4_ and PbCl_2_, the initial reduction peaks were also mapped. For MeP, DQBr_2_, and PQCl_2_, the cathodic peaks were dominant but also some of the smaller anodic peaks were mapped for identification as well. For BPA, several of the anodic peaks clearly played a role in its identification.

## 4. Conclusions

This work demonstrates that multilayer graphene electrodes on a SiC substrate are suitable for electrochemical analysis of seawater using cyclic square wave voltammetry. Machine learning models were applied that are capable of identifying samples based on electrochemical signatures of eight different analytes of interest, ranging from heavy metals to herbicides and pesticides to industrial compounds. Dose-response curves were constructed and used as a basis for quantitative analysis. Future work will focus on deploying the CStat instrument and a graphene electrode for unattended monitoring in maritime regions known for industrial contamination.

## Figures and Tables

**Table 1:** Analysis and fitting parameters of the dose response for each analyte.

Analyte	Peak Potential ^a^	Response ^b^	MSE ^c^	Fitting Parameters			Baseline ^d^	LOD ^e^
				a	b	x_0_		
BPA	E*_pa_* = 0.540	Hyperbolic	74.6	6.9 × 10^−3^	7.2 × 10^−4^	–	0.99	120
CdCl_2_	E*_pa_* = −0.756	Sigmoidal	27.5	36	240	820	1.96	320
CuSO_4_	E*_pa_* = −0.228	Hyperbolic	126.2	4.4 × 10^−3^	2.8 × 10^−4^	–	1.15	140
DQBr_2_	E*_pc_* = −0.536	Hyperbolic	90.3	−0.079	3 × 10^−3^	–	−4.41	130
HgCl_2_	E*_pa_* = 0.108	Linear	38.1	0.0115	0.117		1.39	340
MeP	E*_pa_* = 0.052	Hyperbolic	24.9	3.0 × 10^−4^	4.2 × 10^−4^	–	1.06	380
PbCl_2_	E*_pa_* = −0.568	Sigmoidal	152.7	4.2	170	740	1.37	350
PQCl_2_	E*_pc_*= −0.536	Hyperbolic	38.6	−0.18	6.3 × 10^−3^	–	−4.80	70

^a^ Peak potential vs. Ag/AgCl used for the dose response of analyte; E*_pa_* = anodic peak potential, E*_pc_* = cathodic peak potential; ^b^ Sigmoidal = Equation(1) and Hyperbolic = Equation (2); ^c^ Mean Standard Error; ^d^ Baseline is the average value of current at the peak potential used at zero concentration in μA. ^e^ The limit of detection (LOD) in ppb is estimated using the equation LOD = 3 s/m, where s is the standard deviation of the lowest measureable signal and m is the corresponding slope.
